# Forecasting Nonlinear Chaotic Time Series with Function Expression Method Based on an Improved Genetic-Simulated Annealing Algorithm

**DOI:** 10.1155/2015/341031

**Published:** 2015-04-27

**Authors:** Jun Wang, Bi-hua Zhou, Shu-dao Zhou, Zheng Sheng

**Affiliations:** ^1^National Key Laboratory on Electromagnetic Environmental Effects and Electro-Optical Engineering, PLA University of Science and Technology, Nanjing 210007, China; ^2^College of Meteorology and Oceanography, PLA University of Science and Technology, Nanjing 211101, China

## Abstract

The paper proposes a novel function expression method to forecast chaotic time series, using an improved genetic-simulated annealing (IGSA) algorithm to establish the optimum function expression that describes the behavior of time series. In order to deal with the weakness associated with the genetic algorithm, the proposed algorithm incorporates the simulated annealing operation which has the strong local search ability into the genetic algorithm to enhance the performance of optimization; besides, the fitness function and genetic operators are also improved. Finally, the method is applied to the chaotic time series of Quadratic and Rossler maps for validation. The effect of noise in the chaotic time series is also studied numerically. The numerical results verify that the method can forecast chaotic time series with high precision and effectiveness, and the forecasting precision with certain noise is also satisfactory. It can be concluded that the IGSA algorithm is energy-efficient and superior.

## 1. Introduction

Chaos is a universal complex dynamical phenomenon that exists in various natural and social systems, such as communication, atmosphere, economics, and biology. Chaos phenomenon is generated by determinate equations but the appearance follows an apparently unpredictable nonperiodic stochastic pattern, and it is aperiodic, bounded, deterministic, and sensitive to the initial state. So the prediction of chaotic time series is very useful to evaluate characteristics of dynamical systems and is important to the research of chaos. The prediction of chaotic time series has been widely studied over the years. It is proved that short-term prediction of chaotic time series is possible by exploiting the deterministic dynamics in chaotic systems [1–6]. In general, the forecast of chaotic series implies two processes. The first step is to use the immediate past behavior of the time series to reconstruct state space [7]. Estimating the proper embedding dimensions and delaying time is the main work of state space reconstruction. The self-correlation method [8], mutual information method [9], false nearest neighbors (FNN) algorithm [10], and C-C algorithm [11] have been introduced to reconstruct the state space. The second step is to build the forecasting model. Lots of techniques are proposed to build various models in many literatures, such as artificial neural networks [12–16] and polynomial fitting [17, 18]. Each of them has drawback and advantage; no method is superior to all other methods under every evaluating criterion. The paper proposes a novel and simple predictive model named the function expression method to forecast chaotic time series. In fact, there are many methods to establish the proper function expression. Zhang and Xiao [17] proposed a continued fractions method to give explicit expression. Zhou et al. [18] presented a multivariate local polynomial kernel estimator to approximate polynomial. Although those mathematical methods can predict accurately, they are too complex to predict the complex high dimensional chaotic systems, and the convergence speed is not high enough. The paper proposes an improved genetic-simulated annealing algorithm to establish the best approximation to the real dynamic equation. The genetic algorithm has a strong capability of global optimization and has been used in many forecasting problems [19–23]. However, it is easily trapped into the local-best solution, and the quality of solutions is decreased a lot. We find that the simulated annealing algorithm [24, 25] has strong ability to jump out of the local-best solution and search for the best solution, but the local search ability is relatively poor. Therefore, incorporating the simulated annealing algorithm into the genetic algorithm is an ideal way that combines the global optimization ability of GA with the local search ability of SA; GA is developed to rapidly search for an optimum or near-optimum among the solution space, and then SA is utilized to seek a better one on the basis of that solution. In addition, the fitness function and the genetic operators are also improved to further improve the performance of optimization. The performance of the proposed method is verified by some simulations, and the results demonstrate the outstanding optimization capability and higher forecasting precision compared with other methods such as traditional genetic algorithm (GA) [1], continued fractions (CF) method [17], and neural network (NN) [16].

The remaining sections of this paper are organized as follows. In Section 2, the general forecasting principle of chaotic time series is presented.  Section 3 elaborates the IGSA algorithm and the detailed forecasting procedure. The simulated numerical results are given in Section 4. The effect of noise in the chaotic time series is presented in Section 5. In Section 6, the discussions upon the proposed method are given. The paper ends with conclusions in Section 7.

## 2. General Forecasting Principle

The system state of a chaotic system and its delayed versions can be described in (1), where *t* is a scalar index for the time series, *τ* is the time delay, and *m* is the embedding dimension. Consider

(1) $$\begin{array}{c} \vec{X}\left(\;t\;\right)=\left[\;x\left(\;t\;\right),x\left(\;t-\tau \;\right),\ldots ,x\left(\;t-\left(\;m-1\;\right)\tau \;\right)\;\right]. \end{array}$$

We want to determine the dependence of the state value *x*(*t*) on its previous state values. Takens Embedding Theorem [7] guarantees that the system's state information can be recovered from a sufficiently long observation of the output time series. According to the theorem, the system state follows the existence of a smooth map *T* : *R* ^*m*^ → *R* satisfying

(2) $$\begin{array}{c} x\left(\;t\;\right)=T\left(\;x\left(\;t-\tau \;\right),x\left(\;t-2\cdot \tau \;\right),\ldots ,x\left(\;t-m\cdot \tau \;\right)\;\right). \end{array}$$

Thus, once the state space has been reconstructed from the time series, the chaotic time series can be forecasted by establishing the functional relation *T*(·) with the proposed IGSA algorithm.

Measurements from a chaotic system are not restricted to a unique variable, but situations in which several variables are observed from the same system are common. In this case, we need to deal with multivariate time series {[*x*(*t*_*i*_), *y*(*t*_*i*_),…, *z*(*t*_*i*_)]}, *i* = 1,…, *N*. The model of connection between the different variables can be written in (3), where *H*(·) is the model to be determined. Consider

(3) xt=Hyt−τ,…,yt−m·τ,…,zt−τ,…,zt−m·τ.

**Figure 1:**
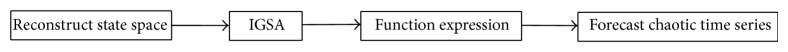
The general procedure of chaotic time series forecasting.

The general principle of using IGSA method to establish the optimum function expression to forecast chaotic series time is shown in Figure 1.

## 3. Function Expression Established by IGSA

In this section, the IGSA algorithm is proposed to establish the functional relation. The genetic algorithm is an evolutionary method that mimics the process of natural selection. GA generates solutions to optimization problems using techniques inspired by natural evolution, such as selection, crossover, and mutation. During each generation, a proportion of the existing population is selected to breed a new generation through a fitness-based process. Crossover is a process of taking more than one parent solution and producing a child solution from them. It is analogous to reproduction and biological crossover. Mutation is used to maintain genetic diversity from one generation of a population of genetic algorithm chromosomes to the next. It is analogous to biological mutation. Mutation alters one or more gene values in a chromosome from its initial state. The purpose of mutation is preserving and introducing diversity. Hence GA can come to better solution by using mutation.

The processes of establishing the best equation by GA can be described as follows. The GA algorithm selects the initial population of potential equations from the initial population of individuals that best fit the real data. The strongest strings choose a mate for reproduction whereas the weaker strings become extinct. The newly generated equations are subject to mutations that change fractions of information. The evolutionary steps are repeated until the optimum equation is established. Although GA can search the optimization solution quickly, the quality of the solution needs to be improved because it is liable to be trapped in local optima. The IGSA algorithm gets over the shortcomings of GA and incorporates the simulated annealing algorithm. The annealing process is usually simulated using a Monte Carlo procedure. In this procedure, the thermal motion of atoms in contact with a heat bath at a given temperature is simulated. The procedure is simply stated here [24]: Given a configuration of the elements of the system, randomly displace the elements on a time by a small amount and calculate the resulting change in the energy, Δ*E*. If Δ*E* < 0 then accept the displacement and use the resulting configuration as the starting point for the next iteration. If Δ*E* ≥ 0 then the displacement is accepted with probability shown in (4), where *T* is the current temperature and *k*_*b*_ is Boltzmann's constant. Repetition of this step continues until equilibrium is achieved. For more detailed information on the simulated annealing, please refer to [24, 25]. Consider the following:

(4) $$\begin{array}{c} P\left(\;\Delta E\;\right)=\exp\left(\;-\frac{\Delta E}{k_{b}T}\;\right). \end{array}$$

**Figure 2:**
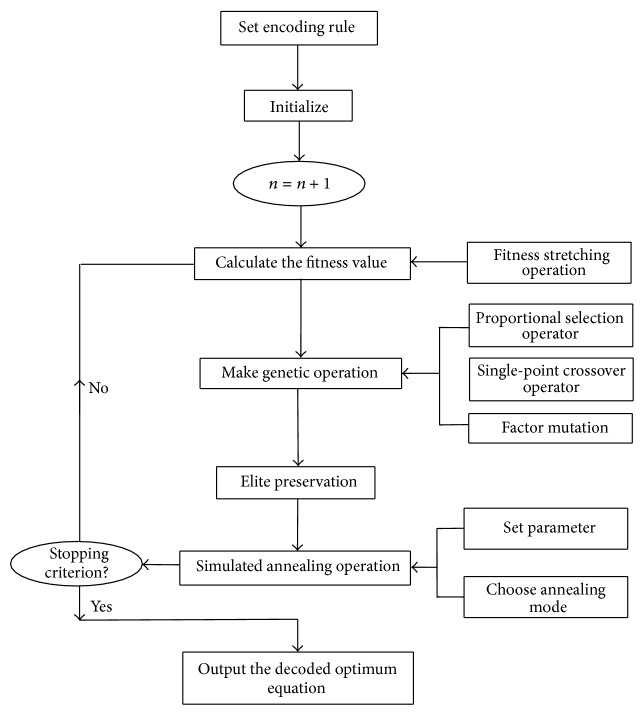
The whole graphical procedure of the proposed IGSA algorithm.

In addition, the fitness function and genetic operators are also improved to further improve the efficiency of the exploration. The whole graphical procedure of the IGSA algorithm is illustrated in Figure 2.

The detailed procedures of using IGSA algorithm to establish the best functional relation are described as follows.

**Step 1 (set the encoding rules).**

In order to encode the symbolic form of the equation strings into a numerical structure, the encoding utilizes coordinate pairs, and the detailed rules are listed in Table 1. The second coordinate indicates an argument or operator in the equation. If the second coordinate is 1, then the pair represents operator. For example, the pair (*p*, 1) represents an operator in the equation string, and the values of *p* range from 1 to 4 to represent the four basic arithmetic operators, addition, subtraction, multiplication, and division, respectively. If the second coordinate is 2, then the pair represents constant term. For example, the pair (*k*, 2) represents the real argument *k* of the equation. If the second coordinate is greater than 2, then the pair represents the variable of the equation. For example, the pair (*i*, 3) represents the element *x*(*t* − *i* · *τ*) in the time series, and (*i*, 4) represents the element *y*(*t* − *i* · *τ*) in the time series.

**Step 2 (initialize).**

The precursor process is the generation of initial population of individuals as a basis for future generations. As the encoding rule of the equation is special, in order to make the decoded individual form the equation, there are several rules when building the initial individuals.

The first two elements of the individual must be constant or variable terms, and the last one must be an operator.

Given one location in the individual the number of nonoperators (constant or variable terms) on the left must be greater than the number of operators.

The total number of nonoperators in the individual must be the total number of operators plus one.

**Step 3 (calculate the fitness value).**

In order to make the difference of the individual fitness obvious, the simulated annealing stretching operation is introduced to calculate the fitness value of individual. The improved fitness value of the *i*-individual is computed as

(5) $$\begin{array}{c} \text{Newfitness}\left(\;i\;\right)=\frac{\exp\left(\text{Fitness}\left(i\right)/T\right)}{\sum_{i=1}^{N}\exp\left(\text{Fitness}\left(i\right)/T\right)}\cdot N, \end{array}$$

where *N* is the total length of the training set, *T* = *G*(*T*_0_), *T*_0_ is the initial temperature, and *G* is the annealing mode. With the iteration going on, the temperature decreases and the superiority of the better individual is reinforced, so that the probability of selecting the better individuals is higher and the probability of selecting the worse individuals is lower. For the short-term predictability of unique and multivariate time series, Fitness(*i*) is computed in (6) and (7), respectively. Consider

(6) Fitnessi1−∑t=m·τ+1Nxt−Tixt−τ,xt−2·τ,…,xt−m−1·τ2∑t=m·τ+1Nxt−1/N−m·τ·∑t=m·τ+1Nxt2,

(7) Fitnessi=1−∑t=m·τ+1Nxt−Hiyt−τ,…,yt−m−1·τ,…,zt−τ,…,zt−m−1·τ2∑t=m·τ+1Nxt−1/N−m·τ·∑t=m·τ+1Nxt2.

**Step 4 (make selection, crossover, and mutation genetic operation).**

The three genetic operations are described in detail as follows.

*The Selection Operation*. The selection operator chooses individuals that are used for crossover and mutation based on the fitness of the individuals. Proportional selection operator is used to select the individuals, and the probability of selecting *i*-individual is proportional to its fitness value. The probability is computed as

(8) $$\begin{array}{c} \text{prob}\left(\;i\;\right)=\frac{\text{Newfitness}\left(i\right)}{\sum_{k=1}^{N}\text{Newfitness}\left(k\right)}. \end{array}$$

*The Crossover Operation*. Once the mates are selected according to their strength, the crossover operation between the two parent individuals is carried out to generate two new offspring. The crossover procedure starts determining randomly one of the arguments, constant terms, or variables terms of the time series in the first individual. If the next element to the right of this randomly determined argument is an operator, then only this argument is considered for interchanging. If the next pair to the randomly selected argument represents another argument, the part of the string used for the crossover is that which is limited between the randomly selected argument and that element of the string where the numbers of nonoperators, *N*_*S*_, and the number of operators, *N*_*O*_, between the randomly selected argument and the element verify the relation *N*_*O*_ + 1 = *N*_*S*_. Notice that the object of this procedure is to interchange self-consistent parts between the equation strings in order to avoid inconsistent mathematical expressions in the offspring. The same operation is carried out for the second equation string. Take the crossover procedure of the following equation strings *F*_1_, *F*_2_ of two parents for example; strings (2,3) and (3.6,2) are the crossover points; the possible pairs of offspring are *F*_1offspring_, *F*_2offspring_, where the bold pairs represent the parts that have been crossed between the parent equations:

(9) F1=1.5,2,1,3,2,3,1,1,3,1,F2=3.6,2,1,3,1,1,2,3,4,1,F1offspring=1.5,2,1,3,3.6,2,1,3,1,1,1,1,3,1,F2offspring=2,3,2,3,4,1.

*The Mutation Operation*. A mutation operation is taken to yield solutions with new information. In order to preserve the information of the top ranked individual, the mutation is not applied to the best individual with the minimal fitness value of the current iteration. Each element of a determined string is changed by a mutation process with some probability. An element is randomly selected and, depending on whether it is a number, a variable, or an operator, if the element is an operator, then the element is self-mutated; if the element is a number or variable, then the element is self-mutated or mutually mutated. Take *F*_2offspring_ for example; the possible mutated string is *F*_2*off*⁡_mut_, where the bold pairs represent the mutations. Consider

(10) $$\begin{array}{c} F_{2\mathrm{off}\_\text{mut}}=\left\{\;\left(\;1,2\;\right),\left(\;1,3\;\right),\left(\;2,1\;\right)\;\right\}. \end{array}$$

**Step 5 (simulated annealing operation).**

After the mutation operation, preserve the best individual of the current *n*th iteration, and compare the fitness Fitness_new of the best individual of the *n*th iteration with the fitness Fitness_old of the best individual of the (*n* − 1)th iteration. If Fitness_new is better than Fitness_old, then replace Fitness_old with Fitness_new and accept the new best individual; the worst individual of current iteration is also replaced with the best individual; otherwise, displacement is accepted with probability shown in (4), and the worst individual of the current iteration is also replaced with the best individual of the current iteration. Set the proper parameters related to SA, and then the local search performance can be well performed to avoid falling into a local optimum.

**Step 6 (determine the best individual of the current iteration and check whether the stopping criterion is met).**

If the criterion is met then decode the optimum individual and obtain the final equation expression; otherwise, continue the iteration processes until the criterion is met or the maximum iteration number is achieved. The decoding rule is defined as follows to assemble the equation strings.

**Figure 3:**
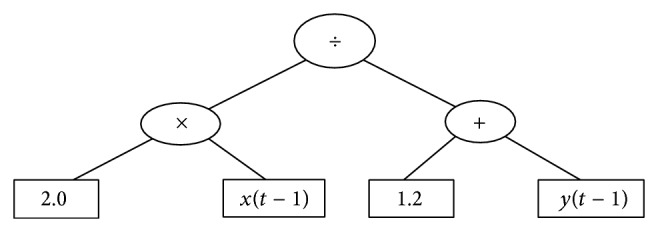
The binary tree structure of the equation strings.

The operator term is placed after its two operands; taking the following coded individual {(2.0,2), (1.0,3), (3,1), (1.2,2), (1,4), (1,1), (4,1)} for example, the decoded equation strings are assembled in the binary tree structure shown in Figure 3. The corresponding equation expression is decoded as (2 · *x*(*t* − 1))/(1.2 + *y*(*t* − 1)).

## 4. Numerical Simulations of Forecasting by the Function Expression

When the optimum function expression is established, the forecasting model is built. In order to verify the feasibility and effectiveness of the prediction of chaotic time series by the function expression, the nonlinear chaotic time series obtained from Quadratic map and Rossler map are adopted as testing objects.

First take the simple unique time series Quadratic map for example. The equation of Quadratic map is

(11) $$\begin{array}{c} x_{n+1}=c-{x_{n}}^{2}, \end{array}$$

where *c* is a constant; here *c* = 1.5, and the initial value is *x*_1_ = 0.8; 400-step values are generated from (11), and the previous 100-step values are abandoned to diminish the influence of the starting value; the 101–300 values are used to train and establish the equation; the remaining 100-step values are taken to test the forecasting performance. The delaying time *τ* and embedding dimension *m* are determined as 1, 2 by use of the method of minimum mutual information [9] and false nearest neighbors algorithm [10], respectively. So the mapping model is

(12) $$\begin{array}{c} x\left(\;n\;\right)=T\left(\;x\left(\;n-1\;\right),x\left(\;n-2\;\right)\;\right). \end{array}$$

The IGSA algorithm is performed to establish the function equation, and the parameters of the algorithm are set as follows. The number of generations *N*_max⁡_ = 5000. The number of initial individuals *N*_pop_ = 100. The length of the individual is 19. The probability of mutation *P*_mut_ = 0.1. The initial temperature *T*_0_ = 1000 (the initial temperature should be set high enough to make the initial accepting probability high). The annealing rate function is the chosen classical annealing function [25] shown in

(13) $$\begin{array}{c} T\left(\;n\;\right)=\frac{T_{0}}{\ln\left(1+n\right)}. \end{array}$$

After iterations the final optimum individual is obtained: {(2.000000,3), (2.000000,3), (1.000000,3), (2.000000,3), (2.000000,3), (2.000000,3), (1.000000,1), (2.000000,1), (2.000000,3), (2.000000,3), (2.000000,3), (1.000000,3), (3.000000,1), (2.000000,1), (4.000000,1), (3.000000,1), (4.000000,1), (2.000000,1), (3.000000,1)}, and the simplified form of the corresponding decoded function expression is

(14) $$\begin{array}{c} x\left(\;n\;\right)=x^{2}\left(\;n-2\;\right)+x\left(\;n-1\;\right)-x^{2}\left(\;n-1\;\right). \end{array}$$

It can be found that *x* ^2^(*n* − 2) + *x*(*n* − 1) = 1.500000 (calculated accurately to sixth place of decimal) from the Quadratic dynamic equation, so that (14) can be written as follows:

(15) $$\begin{array}{c} x\left(\;n\;\right)=1.500000-x^{2}\left(\;n-1\;\right). \end{array}$$

**Figure 4:**
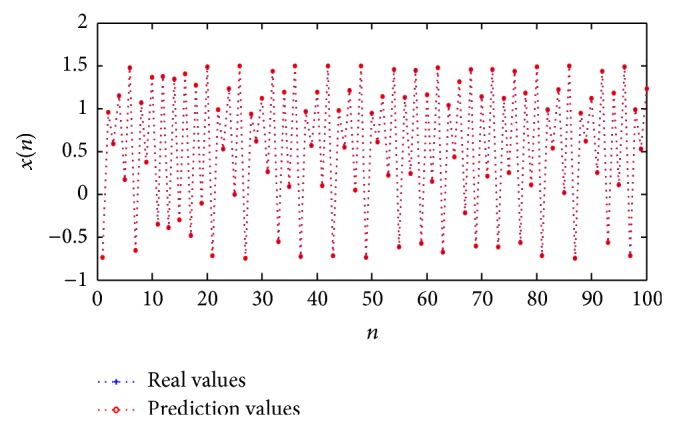
Real values of Quadratic time series and short-term prediction values.

**Figure 5:**
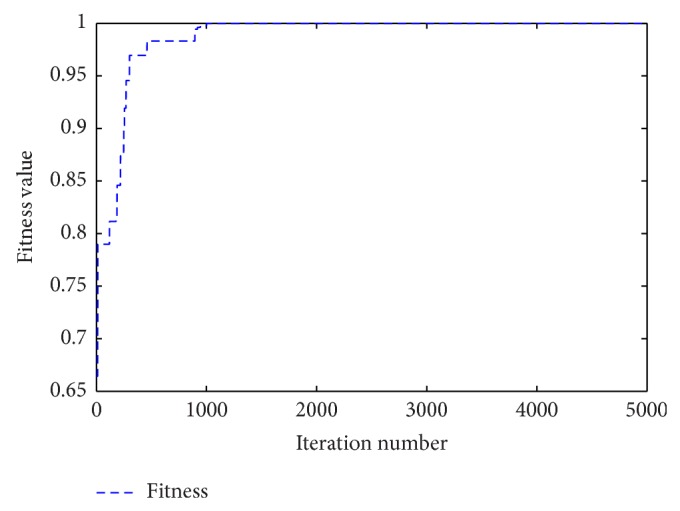
Convergence process of IGSA during the iterations.

It is obvious that (15) is very close to the real map given by (11). Using the independent 100-step values to test the forecasting performance, the real time series generated by (11) and the forecasting time series obtained from (15) are both plotted in Figure 4.  Figure 5 indicates the convergence process of the method of IGSA during the iterations. It can be seen from Figure 4 that the forecasting values are almost the same as the real values. The mean square error (MSE) calculated by (16) is 3.089628*E* − 11, and the value is very close to zero which verifies the high forecast precision of the method. Consider

(16) MSE=1100∑n=301400xn−1.500000−xn−122,

(17) Fitnessbest=1−∑n=301400xn−1.5000000−xn−122∑n=301400xn−1/100·∑n=301400xn2.

Figure 5 indicates that the fitness value converges to the best value with a high speed and the final best fitness value is calculated as Fitness_test_ = 1.000000 (accurately to sixth place of decimal) by (17).

The complex multivariate chaotic Rossler system is also used to test the performance of the proposed method. Rossler map is described as

(18) dxdt−y+z,dydt=x+0.2y,dzdt=0.2+z·x−5.

Time series of all variables (*x*, *y*, *z*) are obtained from solving (18) via 4th-order Runge-Kutta method. The integral step of 4th-order Runge-Kutta method is 0.01, the previous 10000-step values are abandoned, and the following 3000-step values of all variables are adopted to be the sample data, and the 1–2800 values are used to train and establish the equation; the remaining 200-step values are taken to test and validate the forecasting performance. The initial values of the variables are *x*_1_ = −1, *y*_1_ = 0, and *z*_1_ = 1.

Take the *x*-variable for example, and the processes of forecasting *y*-variable and *z*-variable are so similar that they are not given here. The delaying time *τ* and embedding dimension *m* are determined as 1, 4 by use of the minimum mutual information method and false nearest neighbors algorithm, respectively. So the mapping model of *x*-variable is

(19) xt=Hyt−u,…,yt−4·u,…,zt−u,…,zt−4·u.

The parameters of the IGSA method are set as follows. The number of generations *N*_max⁡_ = 5000. The number of initial individuals *N*_pop_ = 120. The length of the individual is 19. The probability of mutation *P*_mut_ = 0.1. The initial temperature *T*_0_ = 1000. The annealing rate function is (13).

The final optimum individual is got after iterations, shown as follows: {(4.914803,2), (−4.694566,2), (2.000000,1), (2.000000,4), (4.000000,4), (2.000000,1), (3.000000,1), (2.000000,4), (4.000000,4), (4.000000,4), (1.000000,1), (2.000000,1), (4.000000,4), (1.000000,5), (1.000000,1), (2.000000,1), (5.713432,2), (4.000000,1), (1.000000,1)}, and the simplified form of the corresponding decoded equation is

(20) xt=9.784395yt−2−10.134447yt−4−0.175026zt−1.

**Figure 6:**
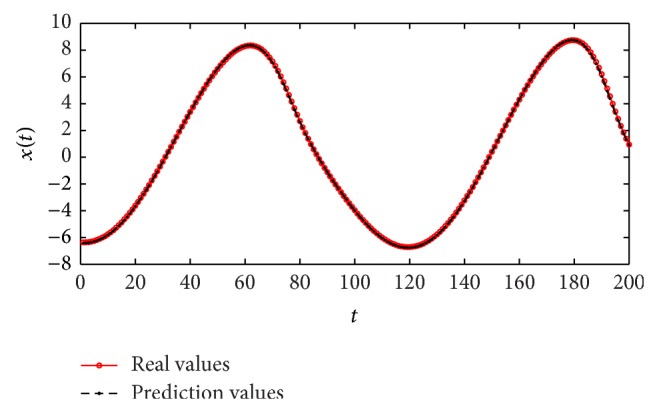
Real values of Rossler time series and short-term prediction values.

**Figure 7:**
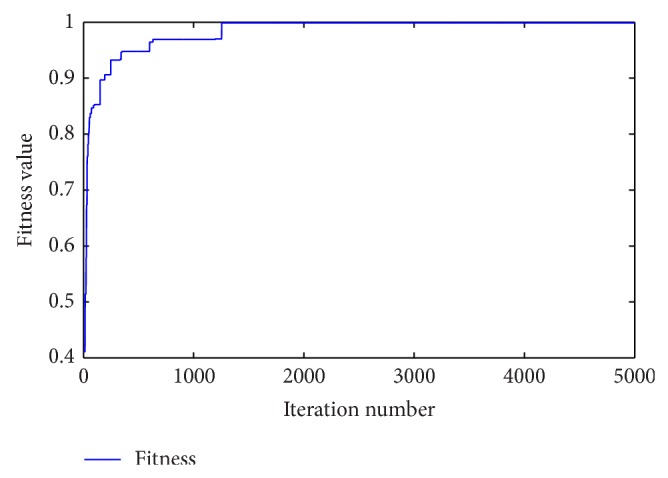
Convergence process of IGSA during the iterations.

It can be seen from (20) that the values of *x*-variable can be extrapolated by the previous values of *y*-variable and *z*-variable. The forecasting performance is verified by taking the remaining 200-step values. The prediction values and real values of Rossler time series are shown in Figure 6, and it can be seen from Figure 6 that the forecasting values are very close to the real values; the mean square error (MSE) is 2.755001*E* − 03.  Figure 7 indicates that the fitness value converges to the best value with a high speed and the final best fitness value is calculated as Fitness_test_ = 0.999903 (accurately to sixth place of decimal).

## 5. Effect of Noise in the Chaotic Time Series

As the actual chaotic systems always contain noise, in order to test the influence of noise on the forecasting precision, the noise-free time series of Quadratic and Rossler maps were corrupted using Gaussian white noise with the different signal-to-noise ratios (SNR) values (see Table 2). The fitness values obtained by taking the same procedure described earlier were also listed in the table.

It can be observed from Table 2 that the fitness values are all decreased, and none of the forecasting precisions matched the precision when noise-free data were used. However, it can be found that the precisions are generally satisfactory when the values of SNR are larger than 25 dB, and when the SNR decreased, the fitness value was decreased a lot. It can be concluded that the proposed method possesses a certain capability of noise immunity and can effectively perform in the noisy time series with large SNR values (in this case, the SNR value should be larger than 25 dB). However, the limit is also obvious that the precisions are not acceptable and the method is not effective in a highly corrupted time series.

## 6. Discussion

In order to demonstrate that the proposed method is robust to parameter variations and to evaluate the effect of parameter variations, different parameters are also set to test the performances of the method, such as the number of generations and initial individuals and the probability of mutation and the initial temperature. The results are varied slightly. However, the following rules of setting the parameters may prove helpful to improve the efficiency and accuracy of the method.

The initial temperature should be set high enough to make the initial accepting probability high. The temperatures from 50 to 3000 (50, 100, 200, 300, 400,…, 3000) are all used to perform the method, and the results indicate that the performance is improved when the temperature increased, but the performance is kept constant until the temperature reaches 1000, so in the paper the temperature is set to be 1000.

The number of initial individuals should be moderate; if the number is too large, then the time consumption is huge; if the number is too small, then diversity of the initial solutions is limited which reduces the quality of solutions.

The number of the generations should also be moderate; if the number is too large, then the time consumption is huge; if the number is too small, then optimal solution may not be searched out during the iterations.

The probability of mutation is typically kept small, and then the diversity of the solutions is improved and the speed of convergence is also kept high. If the probability is large, then the number of iterations increases a lot and the performance is reduced. The probability values from 0.01 to 0.3 are all used to perform the method, and the results are varied slightly with the probability from 0.01 to 0.2. Meanwhile, the performance is reduced when the probability is larger than 0.2.

In order to assess the advantage and improvement of the IGSA method, three representative methods (the traditional genetic algorithm (GA) [1], continued fractions (CF) method [17], and neural network (NN) method [16]) were chosen to compare with the IGSA method. The GA method was applied by Alvarez et al. [1] to forecast chaotic time series; though the precision was high, the accuracy can still be improved a lot as the traditional GA is easily trapped into the local-best solution and the genetic operator can be also improved. The CF method [17] was used by Zhang and Xiao to forecast chaotic time series; it is a pure mathematical method of polynomial approximants, and the main drawback is that it is too complex to predict the complex high dimensional chaotic systems. de Oliveira et al. [16] used the NN method to forecast chaotic systems; the results were almost satisfactory; however, the performance of NN was highly related to the topology structure and initial parameters, and it was easy to fall into local optimal solution.

**Figure 8:**
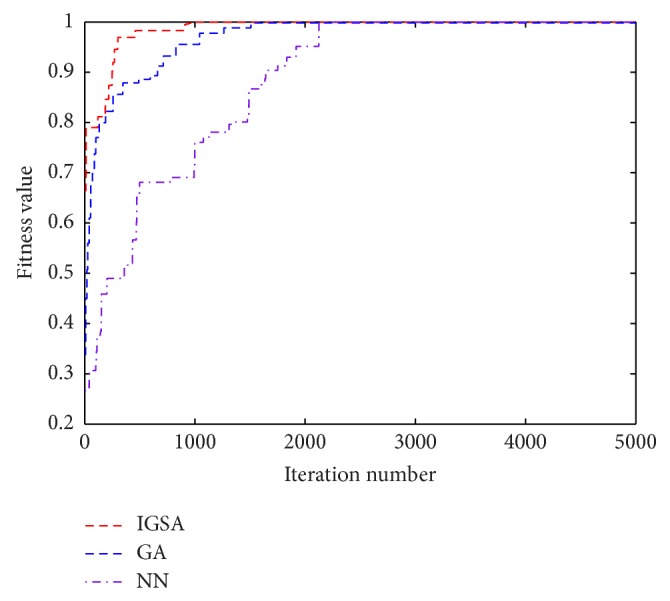
Convergence process of IGSA, GA, and NN methods for Quadratic map during the iterations.

**Figure 9:**
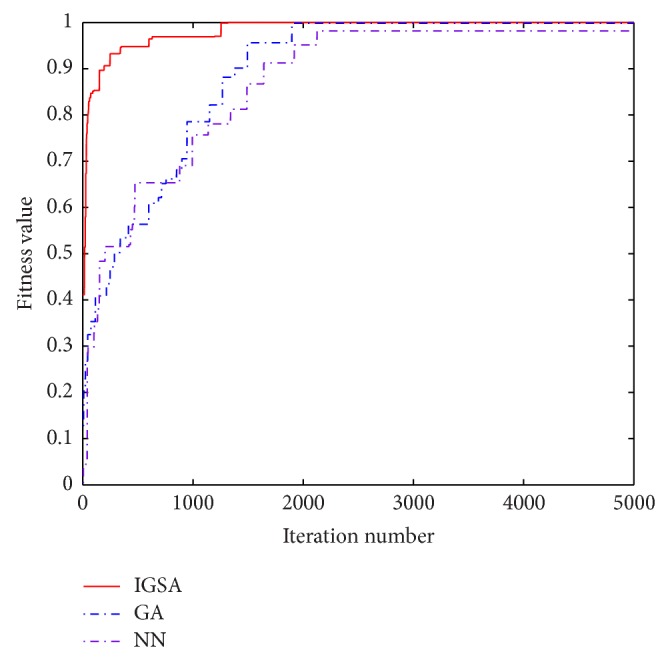
Convergence process of IGSA, GA, and NN methods for Rossler map during the iterations.

The best parameters of NN used to forecast Quadratic and Rossler maps are listed in Table 3 referring to [16], and the ideal architectures of NN are 2-4-2-1 and 4-8-4-1 for forecasting Quadratic and Rossler maps, respectively. The forecasting precisions of Quadratic and Rossler maps evaluated by the different methods are listed in Tables 4 and 5, respectively. The history of convergences for IGSA, NN, and GA is illustrated in Figures 8 and 9.

By the way, Rossler map is too complex to predict by the method of CF, and then the corresponding results are not given here. It can be seen from Tables 3 and 4 and Figures 8 and 9 that the values of fitness and MSE are all satisfactory. However, it is obvious that the results obtained by IGSA are still improved; the fitness value is nearest to 1 and the MSE value is the minimum; besides, the convergence speed is also improved. In fact, it is not easy to further increase the precision when the previous precision is very high; the better values of the fitness value and MSE verifies the superior performance of the proposed algorithm.

Though the proposed method can forecast chaotic time series with high precision and efficiency, the limits are also obvious: The method cannot be applied to the signals which are produced by the complex high dimension systems; it is also unsuitable to apply the method to the signals which are corrupted with low SNR values. Besides, the sample size should be large, and the accuracy and effectiveness of forecasting the chaotic time series with high dimension are decreased; the forecasting precision is decreased when the data are noise-corrupted, and the forecasting results may be ineffective when the SNR values of the noise are not high enough.

## 7. Conclusion

In this paper, we propose a simple but energy-efficient method to predict chaotic time series. The core thought of prediction by the proposed method is adopting an improved genetic-simulated annealing algorithm to construct the optimum function expression to approximate the original dynamic equation of chaotic time series. Then the prediction can be carried out by the constructed expression. The IGSA algorithm incorporates the simulated annealing operation into genetic algorithm to enhance the optimization performance. The simulated annealing stretching operation is introduced to calculate the fitness value and genetic operators are also improved. Finally, two kinds of chaotic time series are used as the testing objects to verify the accuracy and effectiveness of the proposed method, and the corresponding results indicate that performance of forecasting is efficient and satisfactory. The forecast precisions of the simple unique time series and complex multivariate time series are both high enough. In order to evaluate the forecast performance when the system is contaminated with noise, the white noises with different SNR values are added; the results indicate that the method possesses a certain capability of noise immunity and can effectively perform in the noisy time series with large SNR values. We also use other common methods such as traditional genetic algorithm, continued fractions, and neural network to do the same numerical simulations; the compared results demonstrate the obvious superiority and improvement of the proposed method. In general, the proposed IGSA algorithm is a feasible, energy-efficient, and promising method for chaotic time series prediction.

## Figures and Tables

**Table 1:** Encoding rule of equation strings.

	Second coordinate	First coordinate	Pair
Operator term	1	1	+
	1	2	−
	1	3	×
	1	4	÷

Constant term	2	*k* ∈ [−*λ*, *λ*]	*k*

Variable term		1	*x*(*t* − 1 · *τ*)
	3	⋮	⋮
		*i*	*x*(*t* − *i* · *τ*)
		1	*y*(*t* − 1 · *τ*)
	4	⋮	⋮
		*i*	*y*(*t* − *i* · *τ*)
	⋮	⋮	⋮

**Table 2:** Fitness values for noisy time series with different SNR.

	Fitness value							
Chaotic system	SNR value (dB)							
	50	40	35	30	25	20	15	10
Quadratic map	0.9908	0.9012	0.8452	0.8023	0.7473	0.6115	0.4396	0.2618
Rossler map	0.9862	0.8443	0.7629	0.7119	0.6592	0.5361	0.3587	0.2130

**Table 3:** Parameters of NN for forecasting Quadratic and Rossler maps, respectively.

Chaotic system	Parameters of NN				
	Input units number	Hidden layer number	First hidden layer neurons number	Second hidden layer neurons number	Output units number
Quadratic map	2	2	4	2	1
Rossler map	4	2	8	4	1

**Table 4:** Forecasting precisions of Quadratic map calculated by IGSA, GA, CF, and NN methods.

Method	IGSA	GA	NN	CF
Fitness value	1.000000	0.999996	0.999796	0.998845
MSE value	3.089628*E* − 11	2.695395*E* − 06	3.190952*E* − 05	5.522902*E* − 04

**Table 5:** Forecasting precisions of Rossler map calculated by IGSA, GA, and NN methods.

Method	IGSA	GA	NN
Fitness value	0.999903	0.998318	0.981785
MSE value	2.755001*E − *03	4.755993*E − *02	0.514942
